# Graph Adaptive Attention Network with Cross-Entropy

**DOI:** 10.3390/e26070576

**Published:** 2024-07-04

**Authors:** Zhao Chen

**Affiliations:** School of Remote Sensing and Information Engineering, Wuhan University, Wuhan 430081, China; oliver@whu.edu.cn

**Keywords:** non-Euclidean, GCN, adaptive attention mechanism, multi-head graph convolution, cross-entropy

## Abstract

Non-Euclidean data, such as social networks and citation relationships between documents, have node and structural information. The Graph Convolutional Network (GCN) can automatically learn node features and association information between nodes. The core ideology of the Graph Convolutional Network is to aggregate node information by using edge information, thereby generating a new node feature. In updating node features, there are two core influencing factors. One is the number of neighboring nodes of the central node; the other is the contribution of the neighboring nodes to the central node. Due to the previous GCN methods not simultaneously considering the numbers and different contributions of neighboring nodes to the central node, we design the adaptive attention mechanism (AAM). To further enhance the representational capability of the model, we utilize Multi-Head Graph Convolution (MHGC). Finally, we adopt the cross-entropy (CE) loss function to describe the difference between the predicted results of node categories and the ground truth (GT). Combined with backpropagation, this ultimately achieves accurate node classification. Based on the AAM, MHGC, and CE, we contrive the novel Graph Adaptive Attention Network (GAAN). The experiments show that classification accuracy achieves outstanding performances on Cora, Citeseer, and Pubmed datasets.

## 1. Introduction

**Figure 1:**
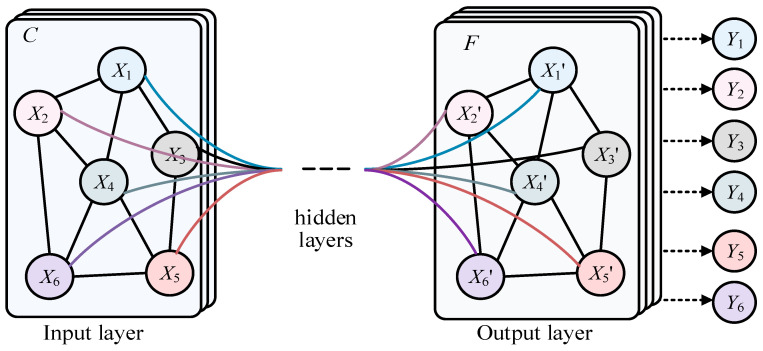
The structure of GCN.

Much data in real life have irregular spatial structures, known as non-Euclidean data, such as social networks, recommendation systems, citation relationships between documents, transportation planning, and natural language processing. This type of data has both node information and structural information, which traditional deep learning networks like the CNN, RNN, and Transformer cannot represent well. The Graph Convolutional Network (GCN) [1], shown in Figure 1, is a class of deep learning models used for processing graph data, and significant progress has been made in graph data in recent years. X_1_–X_6_ represent the different nodes in the graph of input layer, and X’_1_–X’_6_ represent the different nodes in the graph of output layer. In addition, there is also the hidden layer. Finally Y_1_–Y_6_ represent the predicted labels of corresponding nodes. In the real world, many complex systems can be modeled as graph structures, such as social networks, recommendation systems, and automatic modulation classification (AMC), of underwater acoustic communication signals [2]. The nodes and edges of these graph data represent entities and their relationships, which is of great significance for understanding information transmission, node classification, graph classification, and other tasks in graph structures. However, compared with traditional regularized data such as images and text, graph data processing is more complex. Traditional Convolutional Neural Networks (CNNs) [3,4] and Recurrent Neural Networks (RNNs) [5,6] cannot be directly applied to graph structures, because the number of nodes and connections in the graph may be dynamic. Therefore, researchers have begun exploring new graph neural network models to effectively process graph data. Graph Convolutional Networks (GCNs) were proposed in this context, making an important breakthrough in graph data. The main idea of GCNs is to use the neighbor information of nodes to update their representations, similar to traditional convolution operations, but on graph structures. By the weighted averaging of neighboring nodes, the GCN achieves information transmission and node feature updates, allowing the model to better capture the local and global structures in the graph. More broadly, Graph Convolutional Networks (GCNs) are a special case of graph neural networks (GNNs).

The previous graph convolution methods have not fully considered the number and importance of neighboring nodes. To solve the above problems, we propose the novel GAAN (Graph Adaptive Attention Network), and our main contributions are in the following two areas:

(1) To generate different weights for each neighbor node of the central node, we design the novel adaptive attention mechanism (AAM).
(2) Based on the AAM, we utilize Multi-head Graph Convolution (MHGC) to model and represent features better.
(3) We adopt a cross-entropy loss function to model the bias between the predicted values and the ground truth, which greatly improves the classification accuracy.

## 2. Related Work

Graph Convolutional Networks (GCNs) have emerged as a powerful tool for deep learning on graph-structured data, demonstrating impressive performance across various domains such as social network analysis, bioinformatics, and recommendation systems. GCN methods can be broadly categorized into two types based on their convolution approach: spectral-based methods and spatial-based methods. In this paper, we synthesize key literature on these two approaches, discussing their principles, advantages/disadvantages, and applications.

### 2.1. Spectral-Based Methods

Spectral-based methods are rooted in spectral graph theory and graph signal processing, leveraging the Laplacian spectrum of graphs for convolution operations. The foundation for this approach can be traced back to Bruna et al. [7], who introduced spectral networks. They utilized the Fourier transform to perform convolutions on graphs, marking the initial foray into spectral methods. Defferrard et al. [8] advanced this concept with ChebNet, a method that uses Chebyshev polynomials to approximate the spectral convolution, significantly enhancing computational efficiency. This method addressed the scalability issue of the original spectral networks by localizing the convolution operation. Kipf and Welling [1] made a seminal contribution with their Semi-Supervised Graph Convolutional Networks (GCNs), simplifying the spectral convolution process with a first-order approximation. This innovation allowed GCNs to operate efficiently on large-scale graph data and established a benchmark in the field. Their work demonstrated the practical applicability of spectral methods in semi-supervised learning tasks.

Hammond et al. [9] extended the theoretical foundations of spectral methods by exploring wavelet transforms on graphs. This work enriched the theoretical landscape and provided new tools for signal processing on graphs. Henaff et al. [10] further showcased the potential of spectral methods in handling complex graph structures by applying deep convolutional networks to graph data.

Levie et al. [11] introduced CayleyNets, utilizing Cayley polynomials to increase the flexibility and expressive power of spectral convolutions. Their work highlighted the adaptability of spectral methods to various graph structures and provided a robust framework for further development. Shuman et al. [12] offered a comprehensive overview of signal processing on graphs, systematically explaining the theoretical underpinnings of spectral methods.

Bianchi et al. [13] proposed ARMA-GNN, which employs Autoregressive Moving Average (ARMA) filters to improve the performance of spectral convolutions. This method demonstrated the potential of integrating classical signal processing techniques with deep learning on graphs. Defferrard et al. [14] explored the application of deep networks on toric graphs, illustrating the adaptability of spectral methods to specialized graph structures. Finally, Chung and Hu [15] provided the mathematical foundation for spectral methods with their work on spectral graph theory.

### 2.2. Spatial-Based Methods

Spatial-based methods define convolutions directly on the graph nodes and their neighborhoods, circumventing the computational complexity associated with spectral transformations. Hamilton et al. [16] designed GraphSAGE, a seminal spatial-based method that uses the sampling and aggregation of neighbor node features for efficient node representation learning on large-scale graph data. This approach highlighted the practicality of spatial methods in real-world applications where scalability is crucial.

Veličković et al. [17] made significant strides with the Graph Attention Network (GAT), incorporating attention mechanisms to assign different weights to neighbor nodes based on their importance. This innovation enhanced the expressive power of spatial methods, enabling more nuanced and effective learning on graphs.

Monti et al. [18] demonstrated the application of spatial methods to the graph matrix completion problem with their Geometric Matrix Completion method. This work showcased the versatility of spatial methods in addressing various graph-related tasks. Monti et al. [19] further proposed the Mixture Model Network (MoNet), contriving a general framework for defining convolutional operations on graphs using a mixture model paradigm. This method provided a flexible and powerful tool for graph convolution, accommodating a wide range of graph structures.

Wu et al. [20] designed the Simplified Graph Convolutional Network (SGC), a method that reduces the complexity of traditional GCNs by removing the non-linear activation functions between layers. This simplification not only improved computational efficiency but also retained competitive performance in various tasks, emphasizing the potential of streamlined spatial methods.

Xu et al. [21] addressed the challenge of capturing higher-order dependencies in graphs with their Jumping Knowledge Network (JK-Net). By allowing the network to adaptively select and combine different neighborhood ranges, JK-Net enhanced the capability of spatial methods to learn from complex graph structures.

The Graph Isomorphism Network (GIN) [22] tackled the expressiveness of spatial methods, ensuring that the network can distinguish different graph structures effectively. This method set a new standard for the expressiveness of spatial-based GCNs by drawing on insights from the Weisfeiler–Lehman graph isomorphism test.

Liao et al. [23] presented LanczosNet, which leverages the Lanczos algorithm to improve the efficiency and effectiveness of spatial convolutions. This method demonstrated the potential of integrating numerical optimization techniques with graph neural networks to achieve superior performance.

The development of the Spatial–Temporal Graph Convolutional Network (ST-GCN) [24] extended spatial methods to dynamic graphs, capturing both spatial and temporal dependencies. This expansion opened new avenues for applying GCNs to time-evolving graph data, such as in traffic prediction and action recognition.

Finally, Zhang and Chen [25] introduced the Diffusion Convolutional Neural Network (DCNN), which models the diffusion process on graphs to perform convolutions. This approach provided a novel perspective on spatial methods, emphasizing the importance of modeling the underlying processes governing graph data.

Both spectral-based and spatial-based methods have significantly advanced the field of Graph Convolutional Networks, each offering unique advantages and applications. Spectral methods excel in leveraging mathematical foundations from graph theory and signal processing, providing a robust theoretical framework and powerful tools for graph convolution. Spatial methods, on the other hand, offer practical scalability and flexibility, making them suitable for a wide range of real-world applications. This paper aims to solve practical social network issues, so the research is based on spatial methods. To address the differences in the numbers and contributions of neighboring nodes of central nodes, in this paper, we designed the novel Graph Adaptive Attention Network (GAAN).

## 3. Methodology

### 3.1. Overall

**Figure 2:**
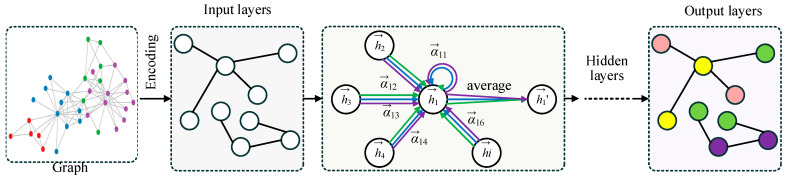
The structure of GAAN. The different color arrows represent Multi-head Graph Convolution (MHGC) and the different color circles represent separate categories of nodes.

The overall structure of the Graph Adaptive Attention Network (GAAN) is illustrated in Figure 2. The process starts with a graph where each node represents an entity and edges denote relationships between them. In the encoding stage, the input graph’s information is transformed into features for each node. The input layer processes these encoded features. Within the graph layer and hidden layer, the network uses an adaptive attention mechanism (AAM) to assign varying weights αij to the neighbors of a central node hi. These weights signify the importance of each neighboring node hj in contributing to the central node’s updated features. The updated features are computed through an attention-based weighted average of neighboring node features. Finally, the output layers aggregate the refined node features to perform node classification, as illustrated in the output graph with colored nodes. 

### 3.2. Graph Layers with AAM

The specific computational process can be represented by Equations (1)–(5).

(1) $$h_{ij}=concat\left(W{\vec{h}}_{i},W{\vec{h}}_{j}\right),\;\;i,j\in \;N$$

where ${\vec{h}}_{i}$ and ${\vec{h}}_{j}$ represent the ith and jth nodes in the graph, respectively. W is the shared weight matrix to unify the node features. $h_{ij}$ fuses the features of the ith and jth nodes. Based on $h_{ij}$, we can calculate the basic AAM between the ith and jth nodes with Equation (2).

(2) $$e_{ij}=\frac{a*h_{ij}}{\sqrt{{D_{i}*D}_{j}}}$$

where $D_{i}$ and $D_{j}$ represent the degree of the ith and jth nodes, respectively. a is the weight vector to reshape $h_{ij}$. $e_{ij}$ is the weight coefficient between the ith and jth nodes. Then, we adopt the softmax function to normalize $e_{ij}$, as shown specifically in Equation (3).

(3) $$\alpha_{ij}=softmax_{j}\left(e_{ij}\right)=\frac{exp(LeakyReLU\left(e_{ik}\right))}{\sum_{k\epsilon h_{i}}exp(LeakyReLU\left(e_{ik}\right))}$$

Based on the normalized weight coefficients between nodes obtained from Equations (1)–(3), we can perform the graph convolution layer computation, as shown in Equation (4).

(4) $${\vec{h^{\prime }}}_{i}=\sigma (\sum_{j\epsilon N_{i}}\alpha_{ij}W{\vec{h}}_{j})$$

Building on Equation (4), we utilize Multi-head Graph Convolution (MHGC), which involves performing the computation of Equation (4) with MHGC and then averaging these results to obtain the node features of the subsequent layer. The computation process is shown in Equation (5).

(5) $${\vec{h^{\prime }}}_{i}=\sigma (\frac{1}{K}\sum_{k=1}^{K}\sum_{j\epsilon N_{i}}\alpha_{ij}^{k}W^{k}{\vec{h}}_{j})$$

Based on the aforementioned formula, we completed the normalized weight coefficients and inter-layer computation processes for the GAAN. We designed a single hidden layer, thus constructing the GAAN with a total of two layers. 

### 3.3. Cross Entropy Loss 

In the node classification task, our goal is to correctly categorize each node into predefined categories. Suppose our model outputs the predicted probability that node $v_{i}$ belongs to each category as ${\hat{y}}_{i}$ and the true category label as $v_{i}$, where ${\hat{y}}_{i}\in R^{C}$ and $y_{i}\in R^{C}$, C is the number of categories, and N is the number of nodes. The cross-entropy loss function is defined as Equation (6).

(6) $$L=-\frac{1}{N}\sum_{i=1}^{N}\sum_{i=1}^{C}y_{i,c}\log({\hat{y}}_{i,c})$$

where N is the total number of nodes. C is the number of categories. $y_{i}$ represents the true category distribution of node $v_{i}$. ${\hat{y}}_{i}$ is the predicted category distribution.

The derivation process of the cross-entropy loss function is as follows. At the final output layer of the network, the feature representation $h_{i}$ of each node $v_{i}$ will pass through a fully connected layer and the softmax function will be applied to generate the predicted probability distribution. The model output will be the predicted probability distribution of the node ${\hat{y}}_{i}$. It is described in Equation (7).

(7) $${\hat{y}}_{i,c}=\frac{\exp(h_{i}\cdot W_{c}+b_{c})}{\sum_{c^{\prime }=1}^{C}\exp(h_{i}\cdot W_{c^{\prime }}+b_{c^{\prime }})}$$

where $W_{c}$ and $b_{c}$ are the weight and bias of the category, respectively. $h_{i}$ is the feature representation of node $v_{i}$.

The cross-entropy loss measures the difference between the true category distribution and the predicted probability distribution. For each node $v_{i}$, the loss is defined as Equation (8).

(8) $$L_{i}=-\sum_{c=1}^{C}y_{i,c}\log({\hat{y}}_{i,c})$$

To measure the categorization performance over the whole graph, the average loss over all nodes needs to be calculated as Equation (6).

Specific processes could be described as the following four steps.

(1) Encoding features: encode the node features of the input graph to obtain the initial feature representation of the nodes.
(2) Attention mechanism: in the hidden layer, use the attention mechanism to weigh the average of neighboring nodes to obtain the updated node feature representation $h_{i}^{\prime }$.
(3) Fully connected layer: in the output layer, the updated node feature representation is transformed into a fully connected transformation and the softmax function is applied to obtain the predicted probability ${\hat{y}}_{i}$.
(4) Calculate the loss: use the cross-entropy loss function to calculate the difference between the true category distribution $y_{i}$ and the predicted probability distribution ${\hat{y}}_{i}$, and the average to obtain the overall loss L.

Through the above process, we can effectively classify the nodes in the graph and optimize the network parameters through backpropagation to achieve the accurate classification of nodes. 

## 4. Experiments 

### 4.1. Datasets

Cora, Citeseer, and Pubmed have widely used citation network datasets in graph-based machine learning research. In this paper, we use the Cora, Citeseer, and Pubmed datasets, the specific statistics of which are shown in Table 1. Cora consists of 2708 machine learning publications categorized into 7 classes, with each paper cited by or citing other papers. Each node represents a publication, and edges denote citation relationships. Nodes have 1433 binary word attributes. Citeseer includes 3327 research papers grouped into 6 categories. Similar to Cora, nodes signify publications, and edges indicate citations. Each node has a 3703-dimensional binary word vector representing the presence or absence of specific words. Pubmed contains 19,717 scientific publications from the PubMed database, classified into 3 diabetes-related categories. The nodes represent papers, connected by citation edges. Each node is described by a TF-IDF-weighted word vector from a 500-word dictionary.

### 4.2. Ablation Experiments

As shown in Table 2, we conduct ablation experiments on the Cora dataset. Table 2 shows the accuracies (Accuracy%) achieved by different experimental groups (Group1 to Group4) when applying specific modules (AAM and MHGC) and setting different dimensionalities of node feature vectors in hidden layers (n_hidden = 64, 96, and 128). Group1 did not use either of the two modules but had three different n_hidden settings, with corresponding accuracies of 83.9%, 83.5%, and 83%. Group2 and Group3 used the AAM and MHGC modules, respectively, and displayed their accuracies under different n_hidden settings, with values ranging from approximately 84.6% to 85.1%. AAM and MHGC, respectively, increased by 1% and 1.2%. Group4 combined the use of both the AAM and MHGC modules, achieving the highest accuracies under different n_hidden settings, reaching up to 85.6%. Our GAAN can significantly improve accuracies.

### 4.3. Comparison with Other Methods

Table 3 presents the classification accuracy (%) of various methods on three datasets: Cora, Citeseer, and Pubmed. The methods compared include MLP, SemiEmb, DeepWalk, ICA, Planetoid, Chebyshev, GCN, MoNet, and GAT. GAAN (no MHGC) achieves an accuracy of 84.9% on Cora, 74.3% on Citeseer, and 79.5% on Pubmed. GAAN (with MHGC) further improves the performance, achieving the highest accuracy of 85.6% on Cora, 75.5% on Citeseer, and 80.5% on Pubmed, indicating the effectiveness of integrating MHGC. GAT also shows strong results with 83.7% on Cora, 73.2% on Citeseer, and 79.3% on Pubmed. According to Table 3, we can see that GAAN achieves better performance than other methods on Cora and Citeseer datasets.

## 5. Conclusions and Future Work

We have proposed an AAM and MHGC to construct a GCNA, which solves the differences between neighbor nodes and the central node. The experimental results show that our method is superior in accuracy. The Graph Convolutional Network (GCN) is a powerful deep learning model specifically designed to handle graph-structured data. By extending convolution operations from traditional Euclidean data spaces to non-Euclidean structures such as graphs, the GCN has a wide range of applications in real life. For example, in social network analysis, GCN can help us identify structural patterns within communities and relationships between nodes. In recommendation systems, the GCN can help us understand user behavior and preferences, thereby generating more accurate recommendations. In chemical molecule classification, the GCN can help predict the properties and behaviors of molecules, thereby accelerating the discovery and development of new drugs.

Over-smoothing occurs when multi-layers are stacked, leading to the features of all nodes being almost the same. However, many situations are necessary to capture the features of distant neighbors. Therefore, stacking multiple layers of the GCN is inevitable. The strategy of stacking multi-layers, designed to prevent over-smoothing, is urgent.

## Figures and Tables

**Table 1:** Summary of the datasets used in our experiments.

Dataset	Nodes	Edges	Features per Node	Classes
Cora	2708	5429	1433	7
Citeseer	3312	4723	3703	7
Pubmed	19,717	44,338	500	3

**Table 2:** Ablation experimental results on Cora. ‘n’ and ‘n_hidden’ represent the number of graph convolution heads and the dimensionality of node feature vectors in hidden layers, respectively. “×” represents not including the corresponding module and “√” represents including the corresponding module.

	AAM	MHGC (*n* = 8)	n_hidden			Accuracy (%)
			64	96	128	
Group1	×	×	√	×	×	83.9
	×	×	×	√	×	83.5
	×	×	×	×	√	83
Group2	×	√	√	×	×	84.6
	×	√	×	√	×	85.1
	×	√	×	×	√	84.6
Group3	√	×	√	×	×	84.9
	√	×	×	√	×	84.3
	√	×	×	×	√	84.6
Group4	√	√	√	×	×	84.6
	√	√	×	√	×	85.4
	√	√	×	×	√	**85.6**

**Table 3:** Compared experimental results on Cora, Citeseer, and Pubmed.

Method	Cora	Citeseer	Pubmed
MLP [26]	55.1	46.5	71.4
SemiEmb [27]	59	59.6	71.7
DeepWalk [28]	67.2	43.2	65.3
ICA [29]	75.1	69.1	73.9
Planetoid [30]	75.7	64.7	77.2
Chebyshev [8]	81.2	69.8	74.4
GCN [1]	81.5	70.3	79
MoNet [19]	82.2	_	79.1
GAT [17]	83.7	73.2	79.3
TransGNN [31]	85.1	74.1	**80.7**
GAAN (no MHGC)	84.9	74.3	79.5
GAAN (with MHGC)	**85.6**	**75.5**	80.5

## Data Availability

The Cora dataset is available at https://linqs-data.soe.ucsc.edu/public/lbc/cora.tgz, accessed on 29 June 2020. The CiteSeer dataset is available at https://linqs-data.soe.ucsc.edu/public/lbc/citeseer.tgz, accessed on 29 June 2020. The PubMed dataset is available at https://linqs-data.soe.ucsc.edu/public/lbc/pubmed_diabetes.tgz, accessed on 29 June 2020.
